# Influence of Craniosacral Therapy on Anxiety, Depression and Quality of Life in Patients with Fibromyalgia

**DOI:** 10.1093/ecam/nep125

**Published:** 2011-06-15

**Authors:** Guillermo A. Matarán-Peñarrocha, Adelaida María Castro-Sánchez, Gloria Carballo García, Carmen Moreno-Lorenzo, Tesifón Parrón Carreño, María Dolores Onieva Zafra

**Affiliations:** ^1^La Vega Sanitary District (Andalusian Health Public Service), Department of Physical Therapy, University of Granada, Spain; ^2^Department of Physical Therapy, University of Almería, Spain; ^3^Department of Psychology, University of Granada, Spain; ^4^Department of Neurosciences, University of Almería, Spain; ^5^Department of Nursing and Physical Therapy, University of Almería (UAL), Spain

## Abstract

Fibromyalgia is considered as a combination of physical, psychological and social disabilities. The causes of pathologic mechanism underlying fibromyalgia are unknown, but fibromyalgia may lead to reduced quality of life. The objective of this study was to analyze the repercussions of craniosacral therapy on depression, anxiety and quality of life in fibromyalgia patients with painful symptoms. An experimental, double-blind longitudinal clinical trial design was undertaken. Eighty-four patients diagnosed with fibromyalgia were randomly assigned to an intervention group (craniosacral therapy) or placebo group (simulated treatment with disconnected ultrasound). The treatment period was 25 weeks. Anxiety, pain, sleep quality, depression and quality of life were determined at baseline and at 10 minutes, 6 months and 1-year post-treatment. State anxiety and trait anxiety, pain, quality of life and Pittsburgh sleep quality index were significantly higher in the intervention versus placebo group after the treatment period and at the 6-month follow-up. However, at the 1-year follow-up, the groups only differed in the Pittsburgh sleep quality index. Approaching fibromyalgia by means of craniosacral therapy contributes to improving anxiety and quality of life levels in these patients.

## 1. Introduction

There is an increasing interest in the role of psychological factors in fibromyalgia, and studies have been published on associated psychological variables, psychopathological explanations, assessment instruments and psychological intervention programs [1, 2]. Suhr (2003) considered psychological factors to be important for understanding the subjective and objective cognitive disorders of fibromyalgia patients [3]. Various investigations have centered on the relationship of fibromyalgia with pain, depression, anxiety and quality of life. The Copenhagen declaration in 1992 described psychological patterns frequently associated with fibromyalgia, such as anxiety and depression, and there is a growing interest in this aspect among professionals of different fields [4]. Nevertheless, many authors consider that psychological factors are more frequently the result than the cause of pain and disability in fibromyalgia, and this issue remains controversial [4].

Some symptoms of fibromyalgia are similar to those observed during depression, and antidepressants have been administered to fibromyalgia patients to treat sleep disorders and pain symptoms [4]. Review of the literature on the association between fibromyalgia and depression reveals two divergent research lines. Hudson and others [5] believe that a direct association cannot be established between fibromyalgia and depression, whereas Gruber and others (1996) [6] propose a common etiology for fibromyalgia and depression. Significant differences in psychological state between patients with fibromyalgia and depression were reported in a study on fibromyalgia, pain intensity and duration and psychological alterations; the results of depression and anxiety questionnaires indicated that the somatic expression of depression differed between the two patient groups [7]. The relationship between depression and fibromyalgia remains controversial. Although antidepressants can reduce pain and fatigue in fibromyalgia, the effects of these drugs vary in degree and duration among patients [7].

Various authors have indicated that patients with fibromyalgia are more depressed than healthy controls and that their perception of psychological distress or depression is similar to that of depressed patients [8, 9]. Moreover, levels of psychological distress (depression, anxiety) have been correlated with cognitive findings in both groups of patients (fibromyalgia and depression) [10–12].

Garland [13] observed a higher degree of anxiety in fibromyalgia patients than in healthy controls or other groups of patients with painful disease, for example, rheumatoid arthritis. Anxious individuals usually have a respiratory dysfunction that generates more work in the upper chest, and the resulting minimum diaphragmatic activity may exacerbate symptoms in patients with fibromyalgia or chronic fatigue syndromes. Although anxiety is known to be an immediate symptom of hyperventilation, it is controversial whether or not hyperventilation and anxiety in patients with fibromyalgia result from a broader alteration. In this context, Peter et al. [14] reported that education to reduce the effects of hyperventilation can reduce fibromyalgia symptoms, including pain, fatigue and emotional distress.

Dysfunction of the autonomic nervous system may explain the different clinical manifestations of fibromyalgia. The hyperactive sympathetic nervous system of these patients becomes incapable of responding to different stressing stimuli, which would explain the continuous tiredness and the morning rigidity of these patients [15]. Likewise, incessant sympathetic activity may explain the sleeping disorders, anxiety, pseudo Raynaud's phenomenon, dry syndrome and intestinal irritability [2, 15]. The other defining characteristics of fibromyalgia such as diffuse pain, painful sensitivity to palpation and paresthesia may also be explained by “sympathetically maintained pain". This neuropathic pain is characterized by a perception of pain regardless of the presence of stimuli, accompanied by paresthesias and allodynia, which are characteristic of patients with fibromyalgia [16].

Patients frequently report sleeping disorders as well as depression, and both factors are known to have a strong association with cognitive disruption [17, 18] and to play an important role in the reduced quality of life reported by fibromyalgia patients. There is a high prevalence of sleeping problems in this population. In many cases, pain and fatigue disappear with sleep. However, paradoxically, patients with fibromyalgia awake with intensified muscle rigidity, pain and marked fatigue [19, 20]. Shaver et al. [21] described a vicious circle of pain, poor sleep, fatigue and increased pain in overt fibromyalgia. Bigatti et al. [22] concluded that sleep predicts subsequent pain in these patients but may be related to depression due to pain and physical dysfunction.

The quality of life of patients with fibromyalgia is especially impaired in relation to physical function, intellectual activity and emotional state, influencing their working capacity and social relationships [23]. Backman [24] affirmed that psychosocial factors are related to two dimensions of experience: psychological (cognitive, affective) and social (interacting with others, performing daily activities). According to this author, psychosocial factors influence the perception of pain, which in turn influences psychological wellbeing and social participation.

Various studies have demonstrated the efficacy of biofeedback acupuncture to reduce pain symptoms in fibromyalgia [25–28]. However, we could find no studies that address the effects of manual therapy on the autonomic nervous system or the possible benefit of this type of alternative therapies as a complement to pharmaceutical treatment of hyperautonomic alterations and derived disorders (anxiety and depression). With this background, the objective of this study was to determine the effects of craniosacral therapy on anxiety, depression, pain, sleep quality and quality of life in fibromyalgia patients up to 1-year post-treatment.

## 2. Methods

### 2.1. Setting and Participants

Patients with fibromyalgia syndrome undergoing pharmaceutical therapy were recruited from among members of the Almeria Fibromyalgia Association with clinical records at the Torrecárdenas Hospital Complex (Almeria, Spain). Inclusion criteria were: diagnosis of fibromyalgia (by rheumatology specialist), age of 16–65 years and agreement to attend afternoon therapy sessions. Exclusion criteria were: presence of physical disease, psychological disease, infection, fever, hypotension or skin disorders or respiratory alterations that would limit the application of the treatments.

Out of the 376 patients in the accessible population, 351 were subjected to a randomization procedure to recruit a sample of 119 patients. Out of these 119 patients, 15 were excluded, and the remaining 104 were randomly assigned by means of a balanced stratified assignment to an intervention (*n* = 52) or placebo (*n* = 52) group. The groups were balanced for type of medication received, sex and age, using a stratification system that generates a sequence of letters (from a table of correlatively ordered permutations) for each category and combination of categories. Informed consent was obtained from all participants according to the ethical criteria established in the Helsinki declaration, modified in 2000, for the performance of research projects. In Spain, current legislation for clinical trials is gathered in the Real Decreto 223/2004 February 6, 2004. This project was approved by the research commissions of the University of Almeria and of the Torrecárdenas Hospital Complex (Almeria)-Servicio Andaluz de Salud (Andalusian Healthcare Service).

Twenty-one patients were under treatment with muscle relaxants, 32 with antidepressants, 46 with anxiolytics, 59 with anti-inflammatories, 36 with corticoids and 84 with analgesics.

#### 2.1.1. Measurements

The following instruments were used to measure anxiety, depression and quality of life in study participants:

- Visual analogue scale (VAS) for pain [29]: This scale assesses the intensity of pain and degree of alleviation experienced by the patient (0 = no pain, 10 = unbearable pain) [30].
- Short form-36 health survey (SF-36) for quality of life: The SF-36 survey evaluates dimensions of functional state, emotional wellbeing and health. Functional state dimensions are: physical function (10 items), social function (two items), role limitations due to physical problems (four items) and role limitations due to emotional problems (three items); emotional wellbeing dimensions are: mental health (five items), vitality (four items) and pain (two items); and health dimensions are: general health perception (five items) and change in health over time (one item—not included in final score) [31].
- Pittsburgh Sleep Quality Index (PSQI): This questionnaire comprises 24 questions, 19 for subjects and 5 for individuals living with them. It yields scores for: subjective sleep quality, sleep latency, sleep duration, habitual sleep efficiency, sleep disturbance, use of hypnotic medication and daily dysfunction. Each component is scored on a scale of 0 to 3 (0 = no problem, 3 = severe problem), yielding an overall score range of 0–21 [32].
- Assessment of the depression index (Beck depression inventory): The Beck inventory is a self-applied questionnaire of 21 items that assesses a broad spectrum of depressive symptoms. It gives weight to the cognitive component of depression, with symptoms in this area representing around 50% of the total questionnaire score. Out of the 21 items, 15 refer to ecological-cognitive symptoms, and six to somatic-vegetative symptoms [33]. The score for each item ranges from 0–3 (from least to greatest severity), giving an overall score range of 0–63 points [34].
- State Trait Anxiety Inventory (STAI): This 40-item questionnaire measures trait anxiety and state anxiety. For the trait anxiety scale (20 items), subjects describe how they feel in general, and for the state anxiety scale (20 items), how they feel at the present time. A score is obtained for each scale [35].

#### 2.1.2. Procedure

In this experimental, longitudinal double-blind clinical trial, the intervention group was formed by 43 patients and the placebo group by 41. Before the treatments, initial assessments of anxiety, depression, pain, sleep and quality of life were performed in all patients [36]. Women of childbearing age were assessed the day after their menstrual period ended. These assessments were repeated at 30 min, six months and 1 year after the last session of the 25-week treatment program.

### 2.2. Intervention

The intervention group underwent a craniosacral therapy protocol, with two weekly sessions of 1 h for 25 weeks. The treatment was carried out by an expert craniosacral therapist with the patient in prone position. This therapy consists of applying very mild manual traction on cranial bones in flexion or extension stages of the craniosacral cycle. The aims were to contribute to re-establishing the normal movement of cranial bones and to intervene in the autonomic nervous system by releasing bone and membranous restrictions [37]. Craniosacral therapy procedures were: still point (occipital), compression-decompression of temporomandibular joint, decompression of temporal fascia, compression-decompression of sphenobasilar joint, parietal lift, frontal lift, scapular waist release and pelvic diaphragm release [37–40].

The placebo group underwent two weekly 30-min sessions of sham ultrasound treatment in which the disconnected probe (4 cm in diameter) was applied to the cervical area (10 min), lumbar region (10 min) and both sides of the knees (10 min). The sham treatment was performed with the patient in prone position. The screen of the ultrasound was covered to ensure that the patient was unaware that the equipment was disconnected.

Both patient groups were instructed not to change their pharmacological treatment during the 25-week study period.

### 2.3. Statistical Analysis

The SPSS package (version 17.0) was used for the data analyses. After performing descriptive statistics of variables at baseline, the Kolmogorov–Smirnof test was applied to evaluate the normal distribution of variables. Continuous data were expressed as means ± SD. A paired *t*-test was used to examine changes in scores between baseline and follow-up examinations. Inter-group differences in variables were analyzed by using repeated-measures analysis of variance. Relationships between demographic variables (sex and age group), aggravating factors, work activity, diseases related to fibromyalgia syndrome, VAS pain score, dimensions of the SF-36 health survey for quality of life, dimensions of the Pittsburgh sleep quality index, total Beck depression inventory score and state and trait anxiety scores were evaluated by calculating Pearson correlation coefficients. A 95% confidence interval (CI) (*α* = 0.05) was considered in all tests.

## 3. Results

**Figure 1:**
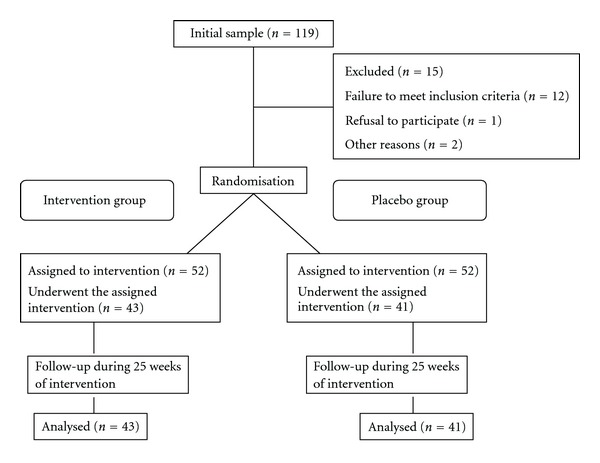
Flow of participants in the study. None of the 84 participants reported adverse effects.

During the study, 9 patients withdrew from the intervention group and 11 from the placebo group. Reasons for withdrawal were death of spouse, start of another type of treatment, change in pharmacologic therapy during treatment period, and missing sessions due to acute pain crisis and forgetfulness. The final study sample comprised 84 patients (81 females) aged 34–63 years with a mean age of 49.08 ± 14.17 years (Figure 1). There were no differences in baseline demographic characteristics between the intervention group (*n* = 43) and placebo group (*n* = 41) (Table 1). The groups did not differ significantly in state anxiety (*P* < .320), trait anxiety (*P* < .269) or VAS (*P* < .239) scores but differed in all dimensions of the SF-36 questionnaire with the exception of vitality. 

In the whole study population, there were significant correlations at baseline between age and physical role (*r* = 0.412; *P* = .008), vitality and general health (*r* = 0.433; *P* = .005), habitual sleep efficiency and social function (*r* = 0.319; *P* = .045) and between mental health and emotional role (*r* = 0.346; *P* = .029), sleep duration (*r* = 0.485; *P* = .001) and habitual sleep efficiency (*r* = 0.328; *P* = .039).

### 3.1. At 35 Weeks after Intervention

**Figure 2:**
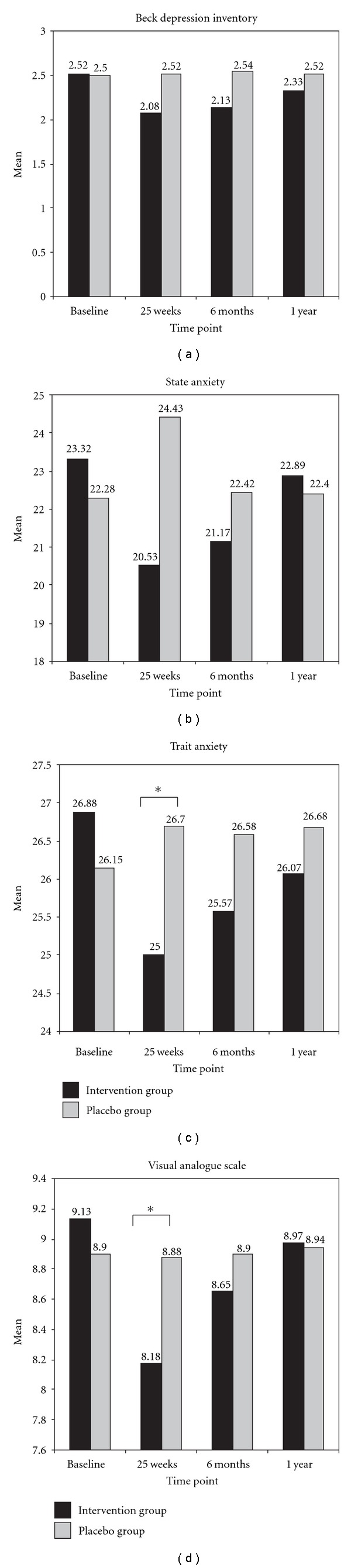
Comparisons between study groups in levels of depression, anxiety and pain. *P = .05 (95% CI). Values are presented as means.

At 35 weeks, the intervention group showed significant improvements in state anxiety (*P* < .029) and trait anxiety (*P* < .042) versus baseline scores. No changes were observed in the placebo group. The groups differed significantly in trait anxiety (*P* < .045). Depression scores did not differ significantly between groups or with respect to baseline values (Figure 2). 

VAS-measured pain improved significantly in the intervention group versus baseline (*P* < .035) and differed between groups (*P* < .041). The intervention group also showed significant improvement in physical function (*P* < .024), physical role (*P* < .020), body pain (*P* < .043), general health (*P* < .039), vitality (*P* < .041) and social function (*P* < .029). The placebo group showed no significant changes versus baseline in SF-36 questionnaire dimensions. The groups differed in physical function (*P* < .009), physical role (*P* < .019), body pain (*P* < .036), general health (*P* < .048), vitality (*P* < .046) and social function (*P* < .028) (Table 2). The intervention group showed a significant overall improvement in Pittsburgh sleep quality index score (*P* < .043), and the groups differed significantly in the sleep duration (*P* < .042) and sleep disturbance (*P* < .040) items (Table 3).

In the intervention group, significant correlations were found between trait anxiety and Beck depression inventory score (*r* = 0.374; *P* = .027), overall SF-36 score and VAS score (*r* = 0.431; *P* = .015), and between physical role and VAS score (*r* = 0.564; *P* = .021), body pain (*r* = 0.378; *P* = .016) and mental health (*r* = 0.385; *P* = .024).

### 3.2. Six Months Post-Intervention

No significant intra-group or inter-group differences were found in state anxiety, depression or pain with respect to baseline. The intervention group showed a significant improvement (versus baseline) in physical function (*P* < .041). The placebo group showed no differences (versus baseline) in any SF-36 questionnaire item. The groups differed significantly in physical function (*P* < .049) and vitality (*P* < .050). The groups also differed significantly in sleep duration (*P* < .039), habitual sleep efficiency (*P* < .047) and sleep disturbance (*P* < .045) (Table 4).

In the intervention group, correlations were found between overall SF-36 questionnaire score and VAS score (*r* = 0.331; *P* = .048) and between trait anxiety score and Beck depression score (*r* = 0.323; *P* = .045).

### 3.3. One Year Post-Intervention

At 1 year, the intervention group showed a significant improvement (versus baseline) in sleep duration (*P* < .040), habitual sleep efficiency (*P* < .044) and daily dysfunction (*P* < .039) (Table 4). No significant differences in anxiety, depression, pain or quality of life were found between groups or with respect to baseline values.

In the intervention group, trait anxiety was correlated with Beck depression score (*r* = 0.311; *P* = .047).

## 4. Discussion

We examined the efficacy of craniosacral treatment on anxiety, depression and quality of life in patients with fibromyalgia. At 6 months after a 25-week treatment period, patients in the intervention group showed a significant improvement in their levels of state anxiety, trait anxiety, pain, quality of life and Pittsburgh sleep quality index.

In comparative studies, patients with fibromyalgia have higher levels of depression in comparison to other patients with chronic diseases. Bennet [41] found that 30% of patients with fibromyalgia present with depression at the first consultation and 60% at some time in their clinical history. These patients reported a diffuse non-localized pain that tended to increase their level of depression.

Recent investigations have not considered depression to be a primary symptom of fibromyalgia, establishing that the degree of depression measured by the Beck questionnaire is closely related to the level of pain suffered by the patient [42, 43]. Nonetheless, a variable percentage of fibromyalgia patients (30–70%) suffer depression, which is also present to some degree in any chronic disease that courses with pain [44]. In multicenter studies, symptoms of major depression appear in 22–68% of patients affected by fibromyalgia, anxiety in 16% and simple phobias in 12–16% [45]. It has not been established whether these psychological disorders are secondary to predominant fibromyalgia symptoms or are primary symptoms of the fibromyalgia syndrome itself, regardless of the remaining symptoms [46–48].

Quality of life results showed a significant post-therapeutic improvement in the physical role, body pain and social function of the intervention group. These findings are consistent with multidisciplinary studies in patients with fibromyalgia, which have underlined the importance of motivation in achieving the participation of patients in the different therapy programs [49–52].

The improvement in physical function achieved by our craniosacral therapy protocol was similar to that obtained by aerobic exercise programs in combination with other exercise modalities and educational programs [53, 54]. Likewise, the improvement obtained in the majority of SF-36 dimensions was similar to that achieved after a 3-month hydrotherapy program, which obtained a 40% reduction in the “body pain" dimension, although the mechanisms underlying this improvement have not been elucidated [55, 56].

The improvement in the SF-36 questionnaire of quality of life shown by intervention group patients was lesser than their improvement in VAS score. This may be explained by the greater sensitivity of the “body pain" dimension of the SF-36 to detect painful changes in comparison to the VAS. Redondo et al. [56] also reported significant differences in the results obtained by these two measures of body pain.

At the end of the treatment period, the intervention and placebo groups differed significantly in overall Pittsburgh subjective sleep quality index score and in habitual sleep efficiency and sleep disturbance items. However, at one month after therapeutic intervention, significant differences were also found in sleep latency and duration. These results are in agreement with those published by Hains and Hains [57], who also found significant differences in sleep quality at one month after a spinal compression and manipulation protocol despite finding no changes in fatigue or pain immediately after the treatment. An improvement in sleep quality persisted for 1 year after a 20-session course of manual therapy involving conjunctive tissue manipulation [58]. The release of fascial restrictions may improve sleep quality by correcting visceral fascial dysfunction and thereby favoring the secretion of platelet serotonin. A study of the gut neurological system found that a high proportion of fibromyalgia patients had intestinal disorders, probably due to neuro-endocrinal causes, which may affect serotonin secretion [59].

Studies on the effects of aerobic exercise programs in fibromyalgia patients found no significant difference in the number of nights per week with sleep disturbances [60–62]. However, multidisciplinary therapeutic programs were reported to significantly improve anxiety, depression, wellbeing and sleep quality [43].

One of the limitations of the study was the inability to study 25 of the 376 patients in the accessible population before the randomized selection of the study group, due to incompatibility with their work schedules. A further limitation is related to the disparity between males and females diagnosed with fibromyalgia, which may be conditioned by the cultural setting. It is also possible that subjects with less severe pain were able to improve more rapidly.

## 5. Conclusions

The present study shows that craniosacral therapy improves the quality of life of patients with fibromyalgia, reducing their perception of pain and fatigue and improving their night rest and mood, with an increase in physical function. Our craniosacral therapy protocol also reduces anxiety levels, partially improving the depressive state. This manual therapy modality must be considered as a complementary therapy within a multidisciplinary approach to these patients, also including pharmaceutical, physiotherapeutic, psychological and social treatments.

## Figures and Tables

**Table 1:** Baseline and demographic characteristics of study groups.

Characteristics	Intervention (*n* = 43)	Placebo (*n* = 41)	*P*
Age mean (SD)	48.25 (13.34)	52.26 (10.98)	.069
Sex (%)			
Female	95.35	97.56	.529
Male	4.65	2.44	.052
Aggravating factors (%)			
Emotional factors	100	100	1.000
Stress	42	50	.086
Work involving standing	46.32	49.48	.194
Cold	100	100	1.000
Work activity (%)			
Full-time	12	18	.098
Part-time	34	26	.067
Sick leave	11	21	.051
Unemployed	43	35	.069
Diseases related to fibromyalgia syndrome (%)			
Arthritis	6.23	8.77	.119
Chorea	2.13	3.87	.201
Type I diabetes	3.25	5.75	.127
Type II diabetes	7	8	.845
Ulcerous colitis	6.5	3.5	.075

*P* = .05 between intervention and placebo groups.

**Table 2:** Differences in quality of life (SF-36 questionnaire) between study groups.

	Baseline *M* (SD)		*P* Pre-T	25 weeks *M* (SD)		*P* 1 ^(a)^-PT	6 months *M* (SD))		*P* 2 ^(a)^-PT	1 year *M* (SD)		*P* 3 ^(a)^-PT
SF-36	IG	PG		IG	PG		IG	PG		IG	PG	
PF	49.43 (6.90)	51.90 (9.92)	.199	45.90 (5.87)	50.53 (9.12)	.009*	46.05 (4.61)	49.05 (8.03)	.049*	47.43 (5.32)	50.68 (7.54)	.367
PR	25.17 (6.88)	25.86 (7.35)	.661	22.10 (6.84)	25.80 (6.98)	.019*	23.85 (7.05)	25.47 (7.09)	.067	24.67 (7.24)	26.01 (7.83)	.121
BP	75.76 (7.20)	78.43 (12.75)	.257	73.12 (6.08)	78.00 (13.07)	.036*	74.25 (6.74)	78.65 (13.22)	.052	74.84 (7.04)	77.39 (10.65)	.234
GH	67.02 (4.25)	68.28 (6.84)	.258	64.40 (4.65)	68.35 (6.39)	.048*	66.02 (4.12)	67.92 (6.69)	.087	66.72 (5.21)	67.63 (7.02)	.321
V	60.05 (5.23)	58.90 (6.27)	.376	62.73 (5.27)	59.48 (7.73)	.046*	60.80 (5.11)	58.72 (7.78)	.050*	61.34 (4.96)	59.01 (5.74)	.201
SF	63.23 (7.12)	63.93 (12.41)	.758	58.75 (6.74)	63.50 (11.57)	.028*	59.85 (10.93)	63.05 (11.87)	.075	60.45 (8.67)	64.45 (10.29)	.067
RE	49.18 (7.65)	46.35 (5.69)	.065	45.60 (7.85)	47.23 (5.66)	.292	49.65 (6.52)	46.40 (5.96)	.053	48.33 (8.31)	47.42 (7.29)	.135
MH	76.65 (11.23)	80.60 (9.66)	.097	77.48 (8.73)	81.15 (10.42)	.069	74.15 (12.12)	77.80 (7.84)	.074	75.64 (9.86)	79.45 (10.35)	.083

Values are presented as means and standard deviations (SD). IG, intervention group; PG, placebo group; Pre-T, pre-therapy; 1 ^(a)^ PT, post-therapy after 25 weeks of treatment; 2 ^(a)^ PT, post-therapy at 6 months after end of treatment; 3 ^(a)^ PT, post-therapy at 1 year after end of treatment; PF, physical function; PR, physical role; BP, body pain; GH, general health; V, vitality; SF, social function; ER, emotional role; MH, mental health. *P = .05 (95% CI).

**Table 3:** Differences between study groups in Pittsburgh sleep quality index score at baseline and after therapy.

	Baseline (*n*)						*P*	25 weeks (*n*)						*P*
	IG			PG			Pre-T	IG			PG			1 ^(a)^-PT
PSQI	NP	MP	SP	NP	MP	SP		NP	MP	SP	NP	MP	SP	
PSQ	0	12	31	2	13	26	.064	0	10	33	5	18	18	.043*
SL	2	6	35	2	7	32	.948	0	22	21	1	12	28	.064
SD	1	12	30	2	5	34	.059	0	19	24	0	10	31	.042*
HSE	1	15	27	0	10	31	.255	0	20	23	0	13	28	.065
SDI	0	17	26	3	14	24	.191	0	27	16	4	10	27	.040*
DD	0	34	9	0	31	10	.592	3	35	5	0	29	12	.065

Values are shown as *n* = number of patients with no problems, moderate problems, severe problems. PSQI, Pittsburgh sleep quality index; IG, intervention group; PG, placebo group; Pre-T, pre-therapeutic; 1 ^(a)^ PT, post-therapy (after 25 weeks of treatment); PSQ, Pittsburgh subjective quality; SL, sleep latency; SD, sleep duration; HSE, habitual sleep efficiency; SDI, sleep disturbance; DD, daily dysfunction; NP, no problems; MP, moderate problems; SP, severe problems. *P = .05 (95% CI).

**Table 4:** Differences between study groups in Pittsburgh sleep quality index at 6 months and 1 year after treatment.

	6 months (*n* = number of patients)						*P*	1 year (*n* = number of patients)						*P*
	IG			PG			2 ^(a)^-PT	IG			PG			3 ^(a)^-PT
PSQI	NP	MP	SP	NP	MP	SP		NP	MP	SP	NP	MP	SP	
PSQ	5	13	25	7	18	16	.093	4	11	28	6	19	16	.054
SL	4	18	21	2	14	25	.105	2	18	23	3	14	24	.132
SD	2	16	25	3	7	31	.039*	1	17	25	2	6	33	.040*
HSE	4	19	20	0	14	27	.047*	2	20	21	0	13	28	.044*
SDI	0	21	22	4	11	26	.045*	0	19	24	2	13	26	.088
DD	6	21	16	0	24	17	.240	3	18	22	1	28	12	.039*

Values are shown as *n* = number of patients with no problems, moderate problems, severe problems. PSQI, Pittsburgh sleep quality index; IG, intervention group; PG, placebo group; Pre-T, pre-therapy; 2 ^(a)^ PT, post-therapy at 6 months after end of treatment; 3 ^(a)^ PT, post-therapy at 1 year after end of treatment; PSQ, Pittsburgh subjective quality; SL, sleep latency; SD, sleep duration; HSE, habitual sleep efficiency; SDI, sleep disturbance; DD, daily dysfunction; NP, does not present problems; MP, moderate problems; SP, severe problems. *P = .05 (95% CI).
